# Paclitaxel-Coated Balloons Versus Sirolimus-Coated Balloons for Coronary Artery Lesions: A Nationwide Cohort Study From SCAAR

**DOI:** 10.1016/j.jscai.2026.105315

**Published:** 2026-04-16

**Authors:** Sacharias von Koch, Troels Yndigegn, Sasha Koul, Sammy Zwackman, Liyew Desta, Oskar Angerås, Elmir Omerovic, Felix Böhm, Ole Fröbert, Per Grimfjärd, Stefan James, David Erlinge, Moman A. Mohammad

**Affiliations:** a Division of Cardiology, Department of Clinical Sciences Lund, Lund University, Lund, Sweden; b Department of Cardiology, Skåne University Hospital, Lund, Sweden; c Department of Health, Medicine and Caring Sciences, Linköping University, Linköping, Sweden; d Department of Cardiology, Linköping University, Linköping, Sweden; e Department of Medicine, Solna, Karolinska Institutet, Stockholm, Sweden; f Department of Cardiology, Karolinska University Hospital, Stockholm, Sweden; g Department of Cardiology, Sahlgrenska University Hospital, Gothenburg, Sweden; h Department of Molecular and Clinical Medicine, Institute of Medicine, Sahlgrenska Academy, University of Gothenburg, Gothenburg, Sweden; i Department of Clinical Sciences, Danderyd University Hospital, Karolinska Institutet, Stockholm, Sweden; j Department of Clinical Medicine, Aarhus University, Denmark; k Department of Cardiology, Faculty of Health, Örebro University, Sweden; l Department of Internal Medicine, Västmanlands Hospital, Västerås, Sweden; m Department of Medical Sciences and Cardiology, Uppsala University and Uppsala Clinical Research Center, Sweden

**Keywords:** drug-coated balloons, paclitaxel-coated balloons, percutaneous coronary intervention, sirolimus-coated balloons, Swedish Coronary Angiography and Angioplasty Registry

## Abstract

**Background:**

Sirolimus-coated balloons (SCB) are a potential alternative to paclitaxel-coated balloons (PCB) in the context of percutaneous coronary intervention (PCI). However, comparative data between SCB and PCB remain limited. We aimed to compare the outcome of PCI with contemporary PCB or SCB in a real-world clinical setting.

**Methods:**

The Swedish Coronary Angiography and Angioplasty Registry (SCAAR) was used to include all patients undergoing PCI with PCB or SCB between January 1, 2022 and May 16, 2025. The primary outcome was target segment revascularization assessed on coronary artery segment level. Outcome was assessed using multivariable-adjusted Cox regression.

**Results:**

A total of 7855 PCB-treated and 502 SCB-treated segments were included. PCB was less frequently used for in-stent restenosis (28.0% vs 34.3%). After a 1-year follow-up period, no difference was observed between PCB and SCB in terms of target segment revascularization (3.3% vs 5.8%; hazard ratio [HR], 1.37; 95% CI, 0.83-2.25), target vessel revascularization (5.2% vs 8.1%; HR, 1.24; 95% CI, 0.78-1.97), restenosis (2.8% vs 4.4%; HR, 1.44; 95% CI, 0.85-2.44), myocardial infarction (3.1% vs 4.9%; HR, 0.74; 95% CI, 0.41-1.32), repeat PCI (8.8% vs 10.8%; HR, 1.04; 95% CI, 0.72-1.51), or all-cause mortality (4.4% vs 3.2%; HR, 1.37; 95% CI, 0.34-1.15).

**Conclusions:**

In this study investigating outcome after PCI with drug-coated balloons, no statistically significant differences in 1-year clinical outcomes were observed between PCB and SCB.

## Introduction

Drug-coated balloons (DCB) enable deposition of an antiproliferative drug to a target coronary segment during percutaneous coronary intervention (PCI), without leaving a permanent scaffold. Previous studies have shown no class effects among DCB and that clinical efficacy may vary between devices.^1^^,^^2^ A key component of the DCB is its antiproliferative drug, but whether the drug efficacy or the device characteristics is the primary determinant of outcome remains unknown. Other important factors include drug dose, balloon design and its excipient. To date, paclitaxel has been the most frequently used drug for coronary DCB.^3^ Paclitaxel works by binding to the β subunit of tubulin resulting in cell cycle arrest.^4^ This drug is lipophilic and quickly absorbed in the tunica media of the artery wall where it has a prolonged tissue retention; at 28 days, arterial levels retained were 9.2% in a coronary porcine model treated with crystalline paclitaxel.^5^ Paclitaxel is a toxic drug that can cause tissue necrosis and, although not clearly understood, there have been reported cases of coronary aneurysm after use of paclitaxel-coated balloons (PCB).^6^, ^7^, ^8^ Also, paclitaxel crystals can flake off, causing distal microembolizations, which may cause damage to the myocardium.^9^ Recently, sirolimus, a mechanistic target of rapamycin inhibitor, has emerged as a potential alternative for DCB.^10^ This drug inhibits smooth muscle cell proliferation without inducing apoptosis. However, the comparative efficacy of sirolimus-coated balloons (SCB) and PCB remains unknown.

In Sweden, 3 SCB are currently available: MagicTouch (Concept Medical), Selution (M.A. Med Alliance SA/Cordis) and Sequent SCB (B. Braun).^3^ MagicTouch failed to demonstrate noninferiority for de novo small vessels in terms of angiographic net lumen gain at 6 months compared to a PCB (SeQuent Please).^11^ When Sequent SCB was compared with the same PCB, similar outcomes were observed in terms of angiographical outcomes; however, the PCB showed more frequent late luminal enlargement.^12^ More recently, the SELUTION DeNovo trial, presented at TCT 2025, showed that a strategy using Selution SCB with provisional stenting achieved a target–vessel failure rate of 5.3% vs 4.4% for a strategy of routine drug-eluting stents, meeting noninferiority.^13^^,^^14^ In parallel, the SELUTION4ISR trial, also presented at TCT 2025, including patients with in-stent restenosis, found a 12-month target–lesion failure rate of 15.2% in the Selution SCB arm vs 13.5% in a standard-of-care arm, satisfying noninferiority.^15^

These studies have marked major milestones for PCI with SCB and reignited the question of class effect among DCB. Although PCB may provide angiographic advantages compared with SCB, consistent differences in hard clinical outcomes have not been clearly demonstrated to date. Previous studies comparing PCB and SCB are limited by small study populations, and most are restricted to comparisons between a single SCB and a single PCB, reducing generalizability. In this real-world registry study, we assessed the impact of the antiproliferative drug by comparing the clinical efficacy of contemporary PCB and SCB in a routine clinical practice in Sweden.

## Methods

### Data source

This is a nationwide retrospective multicenter all-comer study conducted using data from the Swedish Coronary Angiography and Angioplasty Registry (SCAAR). SCAAR is the Swedish PCI registry that encompasses all patients in Sweden who have undergone coronary angiography or PCI in any of the 29 hospitals with a catheterization laboratory. SCAAR includes extensive information on patient characteristics, procedural aspects, and lesion characteristics. To access data on survival status, we merged SCAAR with the National Population Registry using unique Swedish personal identification numbers.^16^ By merging SCAAR and the National Population Registry, complete survival status was ascertained up until May 16, 2025. All other variables, including baseline characteristics, adjustment variables, and other outcome measures, were collected using SCAAR. This study was approved by the Swedish Ethical Review Authority (Dnr 2023-00201-01), and the research was carried out in accordance with the appropriate ethical guidelines. According to Swedish legislation, informed consent is not required for studies based on data from SCAAR. All patients included in SCAAR are informed about their inclusion in SCAAR and are given the opportunity to opt out.

### Study design, study population, and outcomes

**Figure 1:**
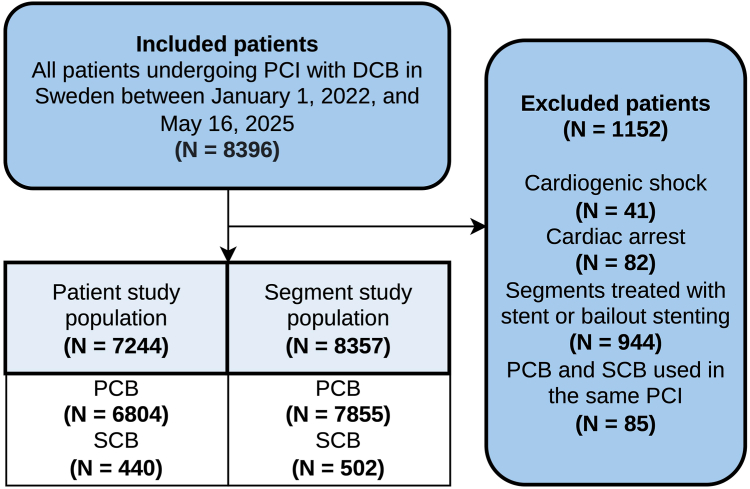
**Flowchart illustrating inclusion and exclusion criteria.** The final study population comprised 7244 patients with 8357 drug-coated balloon (DCB)-treated segments. PCB, paclitaxel-coated balloon; PCI, percutaneous coronary intervention; SCB, sirolimus-coated balloon.

A flowchart with inclusion and exclusion criteria is presented in Figure 1. All patients who underwent PCI with DCB between January 1, 2022, and May 16, 2025, were included. A DCB used for either the culprit lesion or a nonculprit lesion was eligible. Exclusion criteria included cardiogenic shock, cardiac arrest, segments treated with stent or bailout stenting, and use of PCB and SCB within the same procedure. The proportion of segments treated with stent or bailout stenting was 11.2% (944/8396). Thus, no segment requiring bailout stenting was eligible for the analyses. If a patient underwent multiple PCI procedures during the study period, we included only the first PCI for that patient. The day of the PCI was considered the inclusion date. Outcome definitions are presented in detail in Supplemental Table S1. The primary outcome was 1-year target segment revascularization (TSR), defined as a repeat revascularization with PCI in the treated segment. Secondary outcomes included target vessel revascularization (TVR), restenosis, myocardial infarction, repeat PCI, and all-cause mortality. TSR, TVR, and restenosis were analyzed at the segment level, and myocardial infarction, repeat PCI, and all-cause mortality were analyzed at the patient-level. All outcomes were assessed after a 180-day and a 1-year follow-up period. Segment/patients were censored and not considered to be at risk in the case of an event, mortality, completed 1 year of follow-up, or end of the study period (May 16, 2025). All outcome measures were clinically driven events.

### Statistical analysis

Categorical data were analyzed using the χ^2^ test and are presented as counts with percentages. Continuous variables were assessed using *t* test and are presented as means with standard deviation. Outcome was assessed using Cox regression across 4 models: (1) unadjusted, (2) multivariable adjusted, (3) propensity score (PS) adjusted, and (4) PS matched. The unadjusted model included the treatment variable solely. The multivariable model included the treatment variable along with the following variables: inclusion year (2022, 2023, 2024, and 2025), age, previous PCI, stent implantation during the procedure (stent used in another segment), lesion type (de novo, in-stent restenosis, and other restenosis), use of intracoronary imaging, use of fractional flow reserve or instantaneous wave free ratio, lesion preparation (use of cutting balloon, shockwave, and atherectomy), DCB diameter (<2.5 mm, 2.5-3.0 mm, and >3 mm), DCB length (<20 mm, 20-25 mm, and >25 mm), and chronic kidney disease stage (no chronic kidney disease or stage 1 to 2, stage 3, stage 4, and stage 5). Multiple imputations with chained equations were used to impute values for the variable chronic kidney disease stage, given the large number of missing values for this variable (20.9%).^17^ For the PS-adjusted and PS-matched analysis, we used logistic regression to calculate PS. Variables used to calculate PS are presented in Supplemental Table S2. The PS-adjusted model included the treatment variable and the PS. PS matching was conducted using a one-to-one nearest neighbor matching approach with no replacement using a caliper of 0.01. Following PS matching, the outcome was then assessed using univariable Cox regression. The adjustment variables were carefully selected a priori and were based on clinical experience. In addition to the Cox regression analyses, Kaplan-Meier estimates, and log-rank tests were used to assess the outcome. In the segment-level analysis of TSR, TVR, and restenosis, each segment was treated as a unique observation. Considering this methodological approach, we used a multilevel hierarchical model on the patient-level to reduce bias. A subgroup analysis was carried out investigating the outcome separately for de novo lesions and in-stent restenosis. In addition, the following subgroups were analyzed for 1-year TSR using the PS-matched cohort: sex, age (<75 years and ≥75 years), diabetes mellitus, indication (chronic coronary syndrome and acute coronary syndrome), device diameter (≥3.0 mm and <3.0 mm), and device length (≥25 mm and <25 mm). The subgroups were analyzed individually using Cox regression, as well as interaction tests to test for treatment-by-subgroup interactions. For the subgroup analysis, the outcome was assessed using the multivariable Cox regression model. In a supplementary sensitivity analysis, E-values were calculated.^18^ In a second sensitivity analysis, patients were included only up until the 16th of May 2024 to ensure a 1-year complete follow-up for all patients. The proportional hazard assumption was verified using log-log curves and using Schoenfeld residuals for all main Cox models. The global *P* value is presented in Supplemental Table S3. Results are presented as hazard ratios (HRs) with 95% CIs for all Cox regression analyses. All data management and statistical analysis were conducted in STATA version 19.5 (StataCorp LLC).

## Results

### Study population before PS matching

A total of 7244 patients with 8357 DCB-treated segments were eligible (Tables 1 and 2). The PCB group included 6804 patients and 7855 segments, while the SCB group included 440 patients and 502 segments. Patients in the PCB group were more likely to undergo stent implantation during the same procedure (58.6% vs 50.9%). The SCB group had a greater proportion of patients included from 2024 to 2025 and were more likely to have undergone a previous PCI (60.0% vs 54.1%). Segments in the SCB group were more often treated for in-stent restenosis (34.3% vs 28.8%), and intracoronary imaging was more often used during PCI with SCB (28.7% vs 22.3%). The mean follow-up time was 293.3 days (SD, 115.4 days) for the PCB group, 243.2 days (SD, 129.7 days) for the SCB group; 65.7% of the patients had 1 year follow-up available. A list of included DCB is presented in Supplemental Table S4.

**Table 1:** Baseline patient characteristics.

Patient-level characteristics	PCB (n = 6804)	SCB (n = 440)	*P* value
Inclusion year			<.001
2022	1768 (26.0)	39 (8.9)	
2023	1944 (28.6)	100 (22.7)	
2024	2185 (32.1)	184 (41.8)	
2025	907 (13.3)	117 (26.6)	
Age, y	69.6 ± 10.8	71.3 ± 10.3	.002
Age ≥80 y	1277 (18.8)	98 (22.3)	.069
Sex			.60
Male	5293 (77.8)	347 (78.9)	
Female	1511 (22.2)	93 (21.1)	
Smoking status			.48
Nonsmoker	3020 (46.7)	197 (48.9)	
Previous smoker	2685 (41.5)	166 (41.2)	
Active smoker	758 (11.7)	40 (9.9)	
Diabetes mellitus			.45
No diabetes mellitus	4675 (68.7)	294 (66.8)	
Diabetes mellitus without insulin	1235 (18.2)	79 (18.0)	
Diabetes mellitus with insulin	893 (13.1)	67 (15.2)	
Hypertension	5377 (79.0)	347 (78.9)	.93
Hyperlipidemia	4852 (71.5)	300 (68.3)	.16
CKD stage			.60
None or stage 1-2	4464 (82.7)	275 (83.1)	
Stage 3	802 (14.9)	46 (13.9)	
Stage 4	60 (1.1)	3 (0.9)	
Stage 5	70 (1.3)	7 (2.1)	
Previous myocardial infarction	2950 (43.4)	193 (43.9)	.84
Previous PCI	3681 (54.1)	264 (60.0)	.016
Previous CABG	589 (8.7)	42 (9.5)	.52
Indication for PCI			.65
Other	871 (12.8)	52 (11.8)	
Chronic coronary syndrome	1568 (23.0)	109 (24.8)	
Unstable angina	1147 (16.9)	67 (15.2)	
NSTEMI	2239 (32.9)	141 (32.0)	
STEMI	978 (14.4)	71 (16.1)	
Angiographic findings			.65
Atheroma	422 (6.2)	21 (4.8)	
1 VD	2882 (42.4)	186 (42.3)	
2 VD	2017 (29.7)	133 (30.2)	
3 VD and/or LM	1474 (21.7)	100 (22.7)	
Stent implantation	3990 (58.6)	224 (50.9)	.001
Complete revascularization	4916 (73.2)	300 (69.0)	.055
P2Y12 inhibitor			.11
None	208 (3.1)	16 (3.6)	
Clopidogrel	2265 (33.3)	166 (37.7)	
Ticagrelor or prasugrel	4330 (63.6)	258 (58.6)	

Values are n (%) or mean ± SD.

CABG, coronary artery by-pass graft; CKD, chronic kidney disease; LM, left main; NSTEMI, non–ST-segment elevation myocardial infarction; PCB, paclitaxel-coated balloon; PCI, percutaneous coronary intervention; SCB, sirolimus-coated balloon; STEMI, ST-segment elevation myocardial infarction; VD, vessel disease.

**Table 2:** Procedural characteristics

Segment-level characteristics	PCB (n = 7855)	SCB (n = 502)	*P* value
Lesion complexity			.33
A/B1 lesion	3899 (49.6)	238 (47.4)	
B2/C lesion	3956 (50.4)	264 (52.6)	
Bifurcation	1645 (20.9)	121 (24.1)	.093
Graft lesion	129 (1.6)	8 (1.6)	.93
Chronic total occlusion	266 (3.4)	14 (2.8)	.47
Lesion type			.032
De novo	5515 (70.2)	326 (64.9)	
Other restenosis	78 (1.0)	4 (0.8)	
In-stent restenosis	2262 (28.8)	172 (34.3)	
In-stent restenosis type			.157
BMS-ISR	173 (7.7)	11 (6.4)	
DES-ISR	1654 (73.1)	139 (80.8)	
Other ISR	2 (0.1)	0 (0)	
ISR with unknown stent	433 (19.1)	22 (12.8)	
Treated vessel			.45
Left main	117 (1.5)	11 (2.2)	
Left anterior descending	4099 (52.2)	244 (48.6)	
Left circumflex artery	1813 (23.1)	125 (24.9)	
Right coronary artery	1505 (19.2)	99 (19.7)	
Intermediate artery	321 (4.1)	23 (4.6)	
Intracoronary imaging	1754 (22.3)	144 (28.7)	<.001
FFR/IFR	1404 (17.9)	71 (14.1)	.034
Cutting balloon, Shockwave, or atherectomy	511 (6.5)	48 (9.6)	.008
Cutting balloon	427 (5.4)	38 (7.6)	.043
Shock wave balloon	106 (1.3)	11 (2.2)	.12
Atherectomy	6 (0.1)	1 (0.2)	.36
Rotablator	24 (0.3)	4 (0.8)	.065
DCB diameter			.002
<2.5 mm	2277 (29.0)	126 (25.1)	
2.5-3.0 mm	4176 (53.2)	256 (51.0)	
>3.0 mm	1390 (17.7)	120 (23.9)	
DCB length			.22
<20 mm	1987 (25.4)	122 (24.3)	
20-25 mm	3738 (47.7)	227 (45.2)	
>25 mm	2111 (26.9)	153 (30.5)	
Local success	7664 (99.0)	492 (98.6)	.43

Values are n (%).

BMS, bare metal stent; DCB, drug-coated balloon; DES, drug-eluting stent; FFR, fractional flow reserve; IFR, instantaneous wave free ratio; ISR, in-stent restenosis; PCB, paclitaxel-coated balloon; SCB, sirolimus-coated balloon.

### PS-matched study population

Following PS matching, 2 groups of 501 treated segments each were formed. Baseline characteristics were well balanced in terms of patient demographics, comorbidities, lesion characteristics, and procedural aspects (Supplemental Table S5 and Supplemental Figure S1). There were no differences between the 2 groups in any of the variables used to calculate PS. The mean follow-up time was 242.2 days (SD, 134.9 days) for the PCB group and 242.3 days (SD, 129.7 days) for the SCB group.

### Main outcome

**Figure 2:**
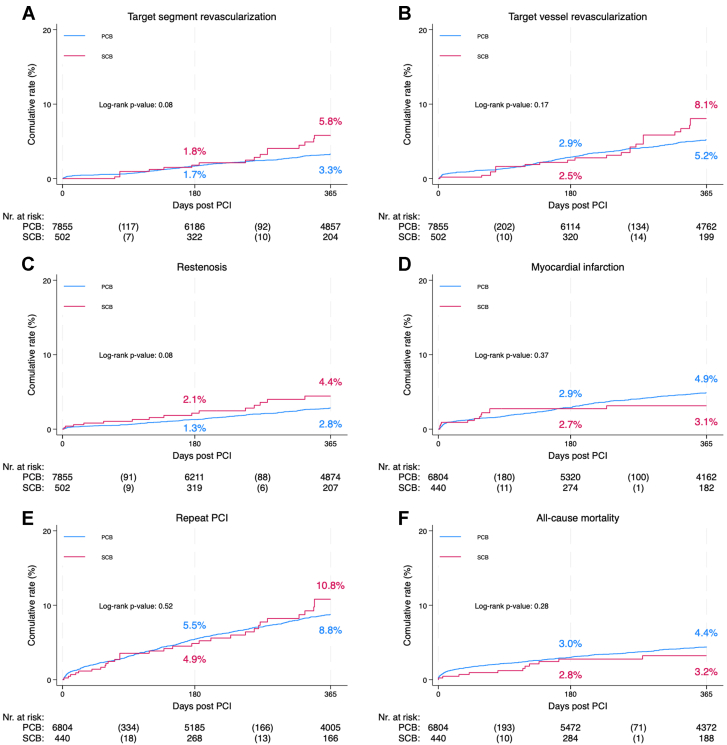
**Kaplan-Meier curves illustrating the number of events at 1 year.** Kaplan-Meier estimates, and log-rank *P* value for (**A**) target segment revascularization, (**B**) target vessel revascularization, (**C**) restenosis, (**D**) myocardial infarction, (**E**) repeat percutaneous coronary intervention (PCI), and (**F**) all-cause mortality. PCB, paclitaxel-coated balloon; SCB, sirolimus-coated balloon.

**Figure 3:**
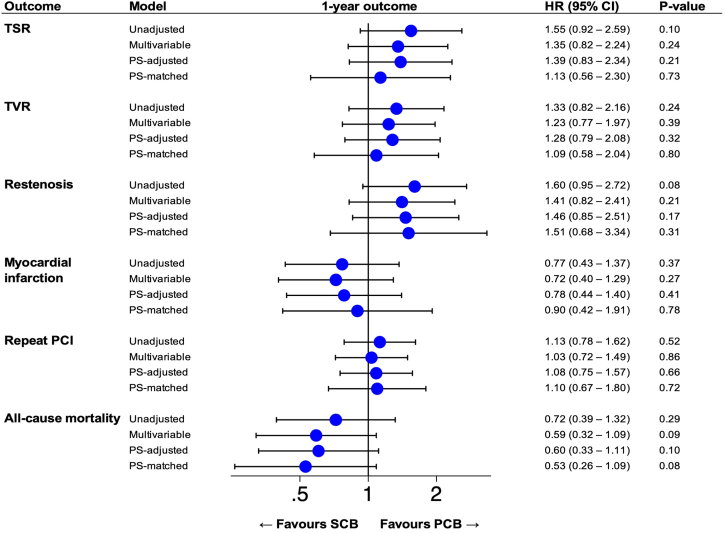
**One-year outcome of target segment revascularization (TSR), target vessel revascularization (TVR), restenosis, myocardial infarction, repeat percutaneous coronary intervention (PCI), and all-cause mortality.** Outcome was assessed using Cox regression across 4 models: unadjusted, multivariable adjusted, propensity score (PS) adjusted, and PS matched. CI, confidence interval; HR, hazard ratio; PCB, paclitaxel-coated balloon; SCB, sirolimus-coated balloon.

After a 1-year follow-up period, no difference was observed between PCB and SCB (Central Illustration). This was observed between PCB and SCB across all of the Cox regression models in terms of TSR (Kaplan-Meier estimates: 3.3% vs 5.8%; multivariable Cox regression: HR, 1.37; 95% CI, 0.83-2.25; *P* = .22), TVR (Kaplan-Meier estimates: 5.2% vs 8.1%; multivariable Cox regression: HR, 1.24; 95% CI, 0.78-1.97; *P* = .37), restenosis (Kaplan-Meier estimates: 2.8% vs 4.4%; multivariable Cox regression: HR, 1.44; 95% CI, 0.85-2.44; *P* = .17), myocardial infarction (Kaplan-Meier estimates: 3.1% vs 4.9%; multivariable Cox regression: HR, 0.74; 95% CI, 0.41-1.32; *P* = .31), repeat PCI (Kaplan-Meier estimates: 8.8% vs 10.8%; multivariable Cox regression: HR, 1.04; 95% CI, 0.72-1.51; *P* = .82) or all-cause mortality (Kaplan-Meier estimates: 4.4% vs 3.2%; multivariable Cox regression: HR, 1.37; 95% CI, 0.34-1.15; *P* = .13) (Figures 2 and 3).

**Central Illustration:**
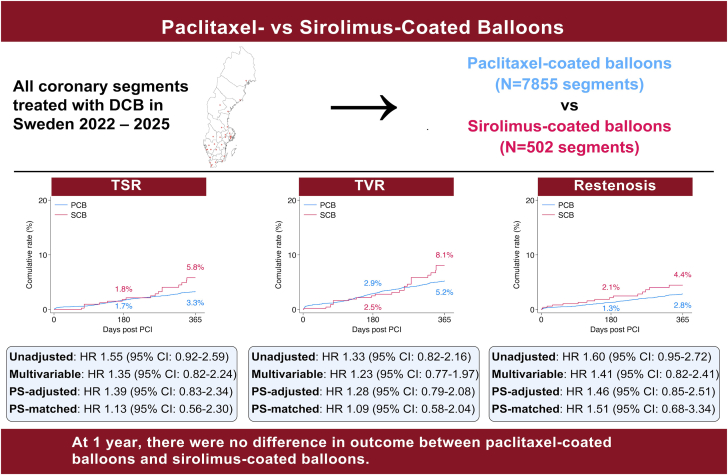
**Paclitaxel-coated balloons vs sirolimus-coated balloons for coronary artery lesions: A nationwide study from SCAAR.** DCB, drug-coated balloons; HR, hazard ratio; PCB, paclitaxel-coated balloons; PS, propensity score; SCAAR, Swedish Coronary Angiography and Angioplasty Registry; SCB, sirolimus-coated balloons; TSR, target segment revascularization; TVR, target vessel revascularization.

### Subgroup and sensitivity analyses

**Figure 4:**
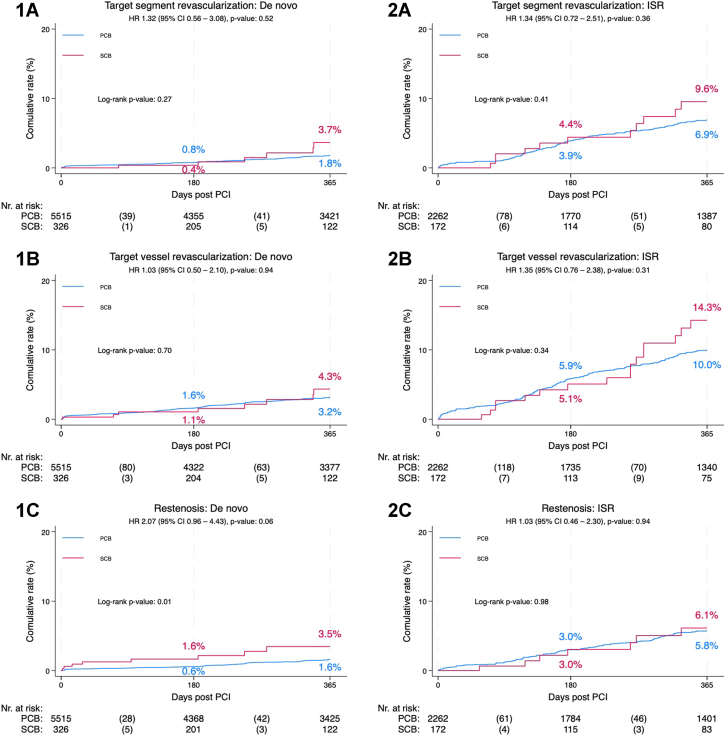
**Subgroup analysis with Kaplan-Meier curves showing outcome for (1) de novo lesions and (2) in-stent restenosis (ISR) for (A) target segment revascularization, (B) target vessel revascularization, and (C) restenosis.** Outcome was assessed using multivariable Cox regression, log-rank test, Kaplan-Meier estimates, and number of events. CI, confidence interval; HR, hazard ratio; PCB, paclitaxel-coated balloon; SCB, sirolimus-coated balloon.

In the subgroup analysis investigating outcome separately for de novo lesions and in-stent restenosis, no difference was observed for any of the subgroups regarding TSR, TVR, or restenosis (Figure 4). No significant treatment-by-subgroup interaction was observed for TSR (*P* value of interaction: 0.918), TVR (*P* value of interaction: 0.651), or restenosis (*P* value of interaction: 0.156). In addition, the following subgroups were analyzed: sex, age (<75 years and ≥75 years), diabetes mellitus, indication (chronic coronary syndrome and acute coronary syndrome), device diameter (≥3.0 mm and <3.0 mm), and device length (≥25 mm and <25 mm). No treatment-by-subgroup interaction was observed among these subgroups (Supplemental Figure S2). No statistically significant difference in 180-day outcome was observed between PCB and SCB (Supplemental Figure S3). When calculating E-values for the 1-year point estimates, E-values ranged between 1.22 and 2.78 (Supplemental Table S6). A sensitivity analysis was conducted, including only patients with 1-year available follow-up; in this analysis, results were in line with the main outcome (Supplemental Figure S4). Finally, in a supplementary analysis adjusting for chronic total occlusion using the PS-matched cohort, the results were in line with the main results, with no statistically significant differences between the two groups (Supplemental Table S7).

## Discussion

In this nationwide all-comer cohort study of patients undergoing PCI with DCB between 2022 and 2025, PCB and SCB had a similar outcome in terms of repeat revascularizations, recurrent myocardial infarctions, and mortality. These findings were consistent for both de novo lesions and in-stent restenosis. To the best of our knowledge, this is the largest study comparing PCB with SCB for coronary lesions.

Differences in the properties of various DCB are undeniable, stemming from variations in manufacturing processes, drug type, coating techniques, excipients, and delivery systems. With growing interest in DCB technology and an expanding evidence base, the question of whether one balloon type outperforms another is becoming increasingly relevant. A key point of interest is whether the choice of drug (paclitaxel or sirolimus) plays a decisive role. PCB remain the most common choice of DCB, accounting for 7854 of 8356 (94.0%) of the DCB in this study. However, recent randomized trials have sparked interest in the use of SCB. In particular, the SELUTION DeNovo trial was a large (N = 3341 patients) multicenter study comparing a strategy with Selution SCB and provisional drug-eluting stents with a systematic use of drug-eluting stents for de novo coronary artery lesions. In this trial, noninferiority was met in terms of target vessel failure (5.3% vs 4.4%; risk difference, 0.91%; *P*_noninferiority_ = .02).^13^^,^^14^ This study will likely accelerate DCB adoption and heighten scrutiny of SCB efficacy relative to PCB. In this context, our study provides valuable real-world data on the comparative efficacy of PCB vs SCB, contributing to a timely and clinically important discussion. In the present study, the event rates of TVR for de novo lesions were 4.3% in the SCB group compared with 3.3% in the SELUTION DeNovo trial. The slightly higher event rate observed in the present study can be explained by the larger proportion of acute coronary syndrome in the present study, as the SELUTION DeNovo trial excluded patients presenting with ST-segment elevation myocardial infarction or unstable non–ST-segment elevation myocardial infarction (63.3% acute coronary syndrome vs 33.3%). When SCB were used for in-stent restenosis, the 1-year event rate of target lesion revascularization was 11.9% in the SELUTION4ISR trial. The subgroup analysis of the present study is in line with this finding, showing a TVR rate of 14.3%, highlighting the challenges with treating in-stent restenosis. A study from the EASTBOURNE registry investigated the use of an SCB (MagicTouch) across de novo lesions and in-stent restenosis.^19^ In this single-arm study, in-stent restenosis was associated with 10.5% target lesion revascularizations at 1 year using SCB. These rates for in-stent restenosis are in line with the present study as well as the SELUTION4ISR trial.

A few studies have investigated the efficacy of SCB compared to PCB (Supplemental Table S8),^11^^,^^12^^,^^20^, ^21^, ^22^, ^23^, ^24^, ^25^, ^26^ and additional randomized clinical trials are currently ongoing.^27^, ^28^, ^29^, ^30^, ^31^ A study combining the EASTBOURNE registry (that included MagicTouch) and PEARL registry (that included Protégé PCB) found no difference in major adverse cardiac events between PCB and SCB across de novo lesions and in-stent restenosis (adjusted HR, 1.17; 95% CI, 0.59-1.23).^25^ The PICCOLETO VI further compared contemporary PCB with SCB for 297 patients with de novo coronary artery lesions.^32^ Compared to the present study, the same SCB were used but with different proportions of each device, including a larger proportion of MagicTouch (MagicTouch n = 51, Selution n = 25, Sequent SCB n = 3), whereas the present study had a larger proportion of Selution and Sequent SCB (MagicTouch n = 18, Selution n = 235, Sequent SCB n = 249). In the PICCOLETO VI study, no statistically significant difference was observed in clinical outcomes at the 1-year follow-up. The results of the present study are in line with the PICCOLETO VI study, showing no difference in clinical outcomes at 1 year. A recently published meta-analysis including 1861 patients treated with DCB found no difference in target lesion failure at 9 to 12 months of follow-up (odds ratio, 1.01; 95% CI, 0.75-1.35); however, the minimal lumen diameter was larger, favoring PCB (weighted mean difference, 0.10; 95% CI, 0.02-0.17).^33^ Although no statistically significant differences were observed in the present study, PCB had numerically lower rates of TSR, TVR, and restenosis, while SCB had numerically lower rates of all-cause mortality. Despite the relatively large sample size, the possibility of a false negative result (type II error) cannot be excluded. Although an even larger cohort or longer follow-up time would have been preferable, this represents the largest study to date addressing this question and provides valuable real-world evidence on the topic.

### Limitations

This study has several limitations; most importantly, DCB use was not randomized. Despite our best attempts to address confounding using multiple adjustment methods, selection bias may still be possible. Although the Swedish national quality registers are known for high data validity, no formal outcome adjudication was performed in this study. As such, some degree of outcome misclassification cannot be ruled out. We consider it likely that any misclassification would be nondifferential concerning the treatment group, which would tend to bias effect estimates toward the null. PS matching was used to reduce confounding by indication. Given unequal treatment prevalence, this resulted in reduced sample size in the PCB group; therefore, unadjusted, PS-adjusted, and multivariable Cox analyses were also performed to complement the PS-matched results. Furthermore, cardiovascular mortality was not available. In this study, the 1-year outcome was assessed, and the results of this study do not confirm that differences between PCB and SCB emerge after long-term follow-up. Segments treated with a stent, for example, bailout stenting, were excluded. This was done to mitigate confounding but may limit the generalizability of the results to segments treated with DCB only. In this study, 7 different types of PCB and 3 different types of SCB were used (Supplemental Table S3). No class effect among DCB exist as device characteristics, drug type, drug dosage, and the excipient influence the efficacy of the DCB. Although extensive information is available through SCAAR, a few important clinical and procedural variables are missing (eg, predilation, final Thrombolysis in Myocardial Infarction [TIMI] flow, residual stenosis, dissection, laboratory values, and discharge medications). Finally, the relatively reduced number of SCB limited the statistical power of the analyses.

## Conclusion

This study found no statistically significant differences in clinical outcomes between PCB and SCB. This held true both in the overall cohort and in stratified analyses of de novo lesions and in-stent restenosis. However, longer-term follow-up studies are warranted to determine whether differences in efficacy may emerge over time.

## Footnotes

To access the supplementary material accompanying this article, visit the online version of the *Journal of the Society for Cardiovascular Angiography & Interventions* at 10.1016/j.jscai.2026.105315.

## References

[1] Bondesson P., Lagerqvist B., James S.K., Olivecrona G.K., Venetsanos D., Harnek J. (2012). Comparison of two drug-eluting balloons: a report from the SCAAR registry. EuroIntervention.

[2] Jeger R.V., Eccleshall S., Wan Ahmad W.A. (2020). Drug-coated balloons for coronary artery disease: third report of the international DCB consensus group. JACC Cardiovasc Interv.

[3] SWEDEHEART SWEDEHEART Annual Report 2025. https://www.ucr.uu.se/swedeheart/dokument-sh/arsrapporter-sh.

[4] Axel D.I., Kunert W., Göggelmann C. (1997). Paclitaxel inhibits arterial smooth muscle cell proliferation and migration in vitro and in vivo using local drug delivery. Circulation.

[5] Granada J.F., Stenoien M., Buszman P.P. (2014). Mechanisms of tissue uptake and retention of paclitaxel-coated balloons: impact on neointimal proliferation and healing. Open Heart.

[6] Hruban R.H., Yardley J.H., Donehower R.C., Boitnott J.K. (1989). Taxol toxicity. Epithelial necrosis in the gastrointestinal tract associated with polymerized microtubule accumulation and mitotic arrest. Cancer.

[7] Farb A., Heller P.F., Shroff S. (2001). Pathological analysis of local delivery of paclitaxel via a polymer-coated stent. Circulation.

[8] Takimura H., Tajima E., Taniguchi R. (2023). Early and late ruptured aneurysm after endovascular therapy with paclitaxel-coated balloon. JACC Cardiovasc Interv.

[9] Xia C., Jiang Y., Li S., Xiong D., Chen X., Chen Y. (2021). In vitro and in vivo comparative evaluation of a shellac-ammonium paclitaxel-coated balloon versus a benchmark device. J Interv Cardiol.

[10] Glotzbecker B., Duncan C., Alyea E., Campbell B., Soiffer R. (2012). Important drug interactions in hematopoietic stem cell transplantation: what every physician should know. Biol Blood Marrow Transplant.

[11] Ninomiya K., Serruys P.W., Colombo A. (2023). A prospective randomized trial comparing sirolimus-coated balloon with paclitaxel-coated balloon in de novo small vessels. JACC Cardiovasc Interv.

[12] Ahmad W.A.W., Nuruddin A.A., Abdul Kader M.A.S.K. (2022). Treatment of coronary de novo lesions by a sirolimus- or paclitaxel-coated balloon. JACC Cardiovasc Interv.

[13] Spaulding C., Krackhardt F., Bogaerts K. (2023). Comparing a strategy of sirolimus-eluting balloon treatment to drug-eluting stent implantation in de novo coronary lesions in all-comers: design and rationale of the SELUTION DeNovo trial. Am Heart J.

[14] Spaulding C., SELUTION DeNovo Investigators (2025). Presented at: TCT 2025; October 26, 2025; San Francisco, CA.

[15] Cutlip D.E., SELUTION4ISR Investigators (2025). Presented at: TCT 2025; October 26, 2025; San Francisco, CA.

[16] Ludvigsson J.F., Otterblad-Olausson P., Pettersson B.U., Ekbom A. (2009). The Swedish personal identity number: possibilities and pitfalls in healthcare and medical research. Eur J Epidemiol.

[17] Kontopantelis E., White I.R., Sperrin M., Buchan I. (2017). Outcome-sensitive multiple imputation: a simulation study. BMC Med Res Methodol.

[18] Chung W.T., Chung K.C. (2023). The use of the E-value for sensitivity analysis. J Clin Epidemiol.

[19] Cortese B., Testa L., Heang T.M. (2023). Sirolimus-coated balloon in an all-comer population of coronary artery disease patients: the Eastbourne prospective registry. JACC Cardiovasc Interv.

[20] Scheller B., Mangner N., Jeger R.V. (2024). A randomised trial of sirolimus- versus paclitaxel-coated balloons for de novo coronary lesions. EuroIntervention.

[21] Liu H., Li Y., Fu G. (2025). Sirolimus- vs paclitaxel-coated balloon for the treatment of coronary in-stent restenosis: the SIBLINT-ISR randomized trial. JACC Cardiovasc Interv.

[22] Pleva L., Kukla P., Kovarnik T., Zapletalova J. (2025). Comparing the efficacy of sirolimus and paclitaxel-eluting balloon catheters in the treatment of coronary in-stent restenosis: a prospective randomized study (TIS 2 study). Circ Cardiovasc Interv.

[23] Zhou Y., Hu Y., Zhao X. (2025). Sirolimus-coated versus paclitaxel-coated balloons for bifurcated coronary lesions in the side branch: the SPACIOUS trial. EuroIntervention.

[24] Cortese B., Caiazzo G., Di Palma G., De Rosa S. (2021). Comparison between sirolimus- and paclitaxel-coated balloon for revascularization of coronary arteries: the SIRPAC (SIRolimus-PAClitaxel) study. Cardiovasc Revasc Med.

[25] Vlieger S., Cheng J.M., Gurgoglione F.L. (2025). Comparison of sirolimus-versus paclitaxel-coated balloons in coronary artery disease: one-year results of two real-world prospective registries. Catheter Cardiovasc Interv.

[26] Leone P.P., Calamita G., Gitto M. (2025). Sirolimus- versus paclitaxel-coated balloons for treatment of coronary artery disease. Am J Cardiol.

[27] (Updated November 22, 2024). Optimal treatment for coronary drug eluting stent in-stent restenosis (OPEN-ISR). ClinicalTrials.gov identifier: NCT04862052. NCT04862052.

[28] (Updated November 29, 2023). ACOART SCB BIF: treatment of coronary bifurcation lesion by sirolimus coated balloon vs paclitaxel coated balloon. ClinicalTrials.gov identifier: NCT04899583. NCT04899583.

[29] (Updated September 17, 2025). Efficacy and safety of sirolimus-coated coronary balloon dilatation catheter for de novo coronary bifurcation lesions. ClinicalTrials.gov identifier: NCT06822712. NCT06822712.

[30] (Updated October 1, 2024). Efficacy and safety of sirolimus-coated spiral balloon for coronary bifurcation lesions. ClinicalTrials.gov identifier: NCT06618248. NCT06618248.

[31] (Updated May 21, 2021). Sirolimus DEB in coronary bifurcation lesions. ClinicalTrials.gov identifier: NCT04896177. NCT04896177.

[32] Fezzi S., Gitto M., Trevisanello A. (2024). TCT-712 Angiographic and angiography-derived functional assessment after DCB angioplasty for de novo coronary artery disease: the PICCOLETO VI study final results. J Am Coll Cardiol.

[33] Shin D., Singh M., Shlofmitz E. (2024). Paclitaxel-coated versus sirolimus-coated balloon angioplasty for coronary artery disease: a systematic review and meta-analysis. Catheter Cardiovasc Interv.

