# Supplementary material for: Paclitaxel-Coated Balloons Versus Sirolimus-Coated Balloons for Coronary Artery Lesions: A Nationwide Cohort Study From SCAAR
Source: J Soc Cardiovasc Angiogr Interv. 2026 Apr 16;5(6):105315. doi: 10.1016/j.jscai.2026.105315 (PMC13404044; doi:10.1016/j.jscai.2026.105315)
Supplement: Supplementary Data [file mmc1.docx]

**Supplemental Appendix**

**Elements included in the supplemental appendix**

**Supplemental Table S1.** Outcome Definition

**Supplemental Table S2.** Adjustment Variables

**Supplemental Table S3.** Schoenfeld Residuals

**Supplemental Table S4.** DCB Included

**Supplemental Table S5.** Baseline Characteristics After PS-Matching

**Supplemental Table S6.** E-values

**Supplemental Table S7.** Adjustment for CTO

**Supplemental Table S8.** Comparative studies of SCB and PCB

**Supplemental Figure S1.** Standardized % Bias Across Covariates

**Supplemental Figure S3.** Subgroup Analysis

**Supplemental Figure S3.** 180-Day Outcome

**Supplemental Figure S4.** Sensitivity Analysis Restricted to Patients with 1-Year Available Follow-Up

**Supplemental Table S1. Outcome Definition**

| **Outcome** | **Source** | **Definition** |
| --- | --- | --- |
| Target segment revascularization | SCAAR | Non-staged repeat revascularization with PCI in the previously treated segment. |
| Target vessel revascularization | SCAAR | Non-staged repeat revascularization with PCI in the previously treated vessel (LAD, RCA, Cx and IM). |
| Restenosis | SCAAR | Significant restenosis (≥50% luminal obstruction) of the previously treated lesion for a patient admitted to coronary angiograph based on a clinical diagnosis. |
| Myocardial infarction | SCAAR | Patient undergoing angiography or PCI due to NSTEMI or STEMI. |
| Repeat PCI | SCAAR | Any non-staged revascularization with PCI. |
| All-cause mortality | National Population Registry | All-cause death. |

Cx = circumflex; IM = intermediary; LAD = left anterior descending; NSTEMI = non-ST-segment elevation myocardial infarction; PCI = percutaneous coronary intervention; RCA = right coronary artery; SCAAR = Swedish Coronary Angiography and Angioplasty Registry; STEMI = ST-segment elevation myocardial infarction.

**Supplemental Table S2. Adjustment Variables**

| **Model** | **Variables** |
| --- | --- |
| Multivariable Cox regression | Inclusion year (2022, 2023, 2024, and 2025), age, previous PCI, stent implantation during the procedure, lesion type (de novo, other restenosis, and in-stent restenosis), use of intracoronary imaging, use of fractional flow reserve or instantaneous wave free ratio, lesion preparation (use of cutting balloon, shockwave or atherectomy), DCB diameter (<2.5 mm, 2.5 – 3.0 mm, and >3 mm), DCB length (<20 mm, 20 – 25 mm, and >25 mm) and chronic kidney disease stage (no chronic kidney disease or stage 1-2, stage 3, stage 4, and stage 5). |
| Variables used to calculate propensity score | Inclusion year (2022, 2023, 2024, and 2025), age, sex, diabetes mellitus (no diabetes mellitus, diabetes mellitus without insulin, and diabetes mellitus with insulin), hypertension, hyperlipidemia, previous myocardial infarction, previous PCI, previous coronary artery by-pass graft surgery, indication (chronic coronary syndrome, NSTEMI, STEMI, and other indications), angiographic findings (atheroma, 1 vessel disease, 2 vessel disease, and 3 vessel disease with/without left main disease), stent implantation, P2Y12 inhibitor (no P2Y12, clopidogrel, and ticagrelor or prasugrel), lesion type (de novo, other restenosis, and in-stent restenosis), lesion location (left main, left anterior descending, left circumflex, and right coronary artery), use of intracoronary imaging, lesion preparation (use of cutting balloon, shockwave or atherectomy), DCB diameter (<2.5 mm, 2.5 – 3.0 mm, and >3 mm), DCB length (<20 mm, 20 – 25 mm, and >25 mm). |

DCB = drug coated balloon; NSTEMI = non-ST-segment elevation myocardial infarction; PCI = percutaneous coronary intervention; STEMI = ST-segment elevation myocardial infarction.

**Supplemental Table S3. Schoenfeld Residuals**

| **Outcome measure** | **Model** | **Global p-value** |
| --- | --- | --- |
| TSR | Unadjusted | 0.020 |
|  | Multivariable | 0.001 |
|  | PS-adjusted | 0.003 |
|  | PS-matched | 0.301 |
| TVR | Unadjusted | 0.002 |
|  | Multivariable | <0.001 |
|  | PS-adjusted | <0.001 |
|  | PS-matched | 0.122 |
| Restenosis | Unadjusted | 0.743 |
|  | Multivariable | <0.001 |
|  | PS-adjusted | 0.316 |
|  | PS-matched | 0.559 |
| Myocardial infarction | Unadjusted | 0.039 |
|  | Multivariable | 0.001 |
|  | PS-adjusted | 0.058 |
|  | PS-matched | 0.011 |
| Repeat PCI | Unadjusted | 0.074 |
|  | Multivariable | 0.081 |
|  | PS-adjusted | 0.004 |
|  | PS-matched | 0.857 |
| All-cause mortality | Unadjusted | 0.902 |
|  | Multivariable | 0.102 |
|  | PS-adjusted | 0.650 |
|  | PS-matched | 0.632 |

PCI = percutaneous coronary intervention; PS = propensity score; TSR = target segment revascularization; TVR = target vessel revascularization.

**Supplemental Table S4. DCB Included**

|  |  | **Complete cohort** |  | **PS-matched cohort** |  |
| --- | --- | --- | --- | --- | --- |
|  | **PCB** | Nr. of treated segments |  | Nr. of treated segments |  |
|  | Braun, SeQuent Please | 3108 |  | 201 |  |
|  | Medtronic, Prevail | 2675 |  | 185 |  |
|  | Boston, Scientific Agent | 1171 |  | 83 |  |
|  | Biotronik, Pantera Lux | 766 |  | 23 |  |
|  | Cardionovum, Restore | 83 |  | 6 |  |
|  | Medtronic, IN.PACT Falcon | 26 |  | 1 |  |
|  | Aachen Resonance, Elutax | 1 |  | 0 |  |
|  | Multiple DCB | 25 |  | 2 |  |
|  | Total | **7855** |  | **501** |  |
|  | **SCB** |  |  |  |  |
|  | Braun, SeQuent SCB | 249 |  | 248 |  |
|  | Cordis, Selution | 235 |  | 235 |  |
|  | Concept Medical, MagicTouch | 18 |  | 18 |  |
|  | Multiple DCB | 0 |  | 0 |  |
|  | Total | **502** |  | **501** |  |
|  |  |  |  |  |  |

DCB = drug-coated balloon; PCB = paclitaxel-coated balloons; PS = propensity score; SCB = sirolimus-coated balloons.

**Supplemental Table S5. Baseline Characteristics after PS-Matching**

| **Patient-level** |  | **PCB** | **SCB** | **p-value** |
| --- | --- | --- | --- | --- |
|  |  | **N=489** | **N=439** |  |
| Inclusion year | 2022 | 49 (10.0%) | 39 (8.9%) | 0.89 |
|  | 2023 | 105 (21.5%) | 100 (22.8%) |  |
|  | 2024 | 200 (40.9%) | 183 (41.7%) |  |
|  | 2025 | 135 (27.6%) | 117 (26.7%) |  |
| Age, mean (SD) |  | 72.0 (10.1) | 71.3 (10.3) | 0.28 |
| Age ≥ 80 years |  | 122 (24.9%) | 98 (22.3%) | 0.35 |
| Sex | Male | 371 (75.9%) | 346 (78.8%) | 0.28 |
|  | Female | 118 (24.1%) | 93 (21.2%) |  |
| Smoking status | Non-smoker | 222 (47.4%) | 197 (49.0%) | 0.89 |
|  | Previous smoker | 199 (42.5%) | 165 (41.0%) |  |
|  | Active smoker | 47 (10.0%) | 40 (10.0%) |  |
| Diabetes mellitus | No diabetes mellitus | 313 (64.0%) | 294 (67.0%) | 0.45 |
|  | Diabetes mellitus without insulin | 103 (21.1%) | 78 (17.8%) |  |
|  | Diabetes mellitus with insulin | 73 (14.9%) | 67 (15.3%) |  |
| Hypertension |  | 390 (79.8%) | 347 (79.0%) | 0.79 |
| Hyperlipidemia |  | 350 (71.6%) | 300 (68.3%) | 0.28 |
| CKD stage | No CKD or stage 1-2 | 320 (83.3%) | 274 (83.0%) | 0.82 |
|  | Stage 3 | 52 (13.5%) | 46 (13.9%) |  |
|  | Stage 4 | 6 (1.6%) | 3 (0.9%) |  |
|  | Stage 5 | 6 (1.6%) | 7 (2.1%) |  |
| Previous myocardial infarction |  | 229 (46.8%) | 192 (43.7%) | 0.34 |
| Previous PCI |  | 300 (61.3%) | 264 (60.1%) | 0.71 |
| Previous CABG |  | 40 (8.2%) | 41 (9.3%) | 0.53 |
| Indication | Other | 68 (13.9%) | 52 (11.8%) | 0.56 |
|  | Chronic coronary syndrome | 100 (20.4%) | 109 (24.8%) |  |
|  | Unstable angina | 80 (16.4%) | 67 (15.3%) |  |
|  | NSTEMI | 160 (32.7%) | 140 (31.9%) |  |
|  | STEMI | 81 (16.6%) | 71 (16.2%) |  |
| Angiographic findings | Atheroma | 28 (5.7%) | 21 (4.8%) | 0.60 |
|  | 1 VD | 200 (40.9%) | 186 (42.4%) |  |
|  | 2 VD | 163 (33.3%) | 133 (30.3%) |  |
|  | 3 VD and/or LM | 98 (20.0%) | 99 (22.6%) |  |
| Stent implantation |  | 249 (50.9%) | 224 (51.0%) | 0.97 |
| Complete revascularization |  | 355 (73.3%) | 300 (69.1%) | 0.16 |
| P2Y12 inhibitor | No P2Y12 inhibitor | 20 (4.1%) | 16 (3.6%) | 0.91 |
|  | Clopidogrel | 179 (36.6%) | 165 (37.6%) |  |
|  | Ticagrelor or Prasugrel | 290 (59.3%) | 258 (58.8%) |  |
| **Segment-level** |  | **PCB** | **SCB** | **p-value** |
|  |  | **N=501** | **N=501** |  |
| Lesion complexity | A/B1 lesion | 232 (46.3%) | 238 (47.5%) | 0.70 |
|  | B2/C lesion | 269 (53.7%) | 263 (52.5%) |  |
| Bifurcation |  | 96 (19.2%) | 121 (24.2%) | 0.055 |
| Graft lesion |  | 10 (2.0%) | 8 (1.6%) | 0.63 |
| Chronic total occlusion |  | 28 (5.6%) | 14 (2.8%) | 0.027 |
| Lesion type | De novo | 341 (68.1%) | 326 (65.1%) | 0.47 |
|  | Other restenosis | 2 (0.4%) | 4 (0.8%) |  |
|  | In-stent restenosis | 158 (31.5%) | 171 (34.1%) |  |
| Lesion location | Left main | 10 (2.0%) | 11 (2.2%) | 0.51 |
|  | Left anterior descending | 268 (53.5%) | 244 (48.7%) |  |
|  | Left circumflex artery | 112 (22.4%) | 125 (25.0%) |  |
|  | Right coronary artery | 84 (16.8%) | 98 (19.6%) |  |
|  | Intermediate artery | 27 (5.4%) | 23 (4.6%) |  |
| Intracoronary imaging |  | 158 (31.5%) | 144 (28.7%) | 0.34 |
| FFR/IFR |  | 69 (13.8%) | 71 (14.2%) | 0.86 |
| Cutting balloon, shockwave or atherectomy |  | 52 (10.4%) | 48 (9.6%) | 0.67 |
| Cutting balloon |  | 42 (8.4%) | 38 (7.6%) | 0.64 |
| Shock wave balloon |  | 16 (3.2%) | 11 (2.2%) | 0.33 |
| Atherectomy |  | 1 (0.2%) | 1 (0.2%) | 1.00 |
| DCB diameter | <2.5 mm | 141 (28.1%) | 126 (25.1%) | 0.45 |
|  | 2.5 mm – 3.0 mm | 253 (50.5%) | 255 (50.9%) |  |
|  | >3.0 mm | 107 (21.4%) | 120 (24.0%) |  |
| DCB length | <20 mm | 140 (27.9%) | 122 (24.4%) | 0.32 |
|  | 20 mm – 25 mm | 206 (41.1%) | 227 (45.3%) |  |
|  | >25 mm | 155 (30.9%) | 152 (30.3%) |  |
| Local success |  | 488 (98.4%) | 491 (98.6%) | 0.79 |

CABG = coronary artery by-pass graft; CKD = chronic kidney disease; Cx = circumflex; DCB = drug-coated balloon; D1 = first diagonal; D2 = second diagonal; FFR = fractional flow reserve; IFR = instantaneous wave free ratio; LAD = left anterior descending; LM = left main; LPD = left posterior descending artery; OM1 = first obtuse marginal; OM2 = second obtuse marginal; RPD = right posterior descending; PCB = paclitaxel-coated balloon; PCI = percutaneous coronary intervention; PLA = posterolateral artery; RCA = right coronary artery; SCB = sirolimus-coated balloon; VD = vessel disease.

**Supplemental Table S6. E-Values**

|  |  |  |  |  |
| --- | --- | --- | --- | --- |
|  |  | **E-value for 1 year HR** |  |  |
|  | TSR | 2.04 |  |  |
|  | TVR | 1.76 |  |  |
|  | Restenosis | 2.17 |  |  |
|  | Myocardial infarction | 2.12 |  |  |
|  | Repeat PCI | 1.22 |  |  |
|  | All-cause mortality | 2.78 |  |  |
|  |  |  |  |  |

HR = hazard ratio; PCI = percutaneous coronary intervention; TSR = target segment revascularization; TVR = target vessel revascularization.**Supplemental Table S7. Adjustment for CTO**

| **Outcome measure** | **HR (95% CI)** | **p-value** |
| --- | --- | --- |
| TSR | 1.16 (0.57-2.33) | 0.683 |
| TVR | 1.09 (0.58-2.04) | 0.782 |
| Restenosis | 1.51 (0.69-3.32) | 0.302 |
| Myocardial infarction | 0.89 (0.42-1.90) | 0.769 |
| Repeat PCI | 1.09 (0.67-1.78) | 0.732 |
| All-cause mortality | 0.51 (0.25-1.05) | 0.069 |

CTO = chronic total occlusion; PCI = percutaneous coronary intervention; TSR = target segment revascularization; TVR = target vessel revascularization.

**Supplemental Table S8. Comparative Studies of SCB and PCB**

| **Previous randomized controlled trials** |  |  |  |  |
| --- | --- | --- | --- | --- |
| **Title** | **Publication year** | **Nr. patients** | **PCB type (manufacturer)** | **SCB type (manufacturer)** |
| Treatment of Coronary De Novo Lesions by a Sirolimus- or Paclitaxel-Coated Balloon | 2022 | 70 | SeQuent Please (B. Braun Melsungen) | SeQuent SCB (B. Braun Melsungen) |
| A Prospective Randomized Trial Comparing Sirolimus-Coated Balloon With Paclitaxel-Coated Balloon in De Novo Small Vessels | 2023 | 121 | SeQuent Please NEO (B. Braun Melsungen) | MagicTouch (Concept Medical) |
| A randomised trial of sirolimus- versus paclitaxel-coated balloons for de novo coronary lesions | 2024 | 70 | SeQuent Please NEO (B. Braun Melsungen) | SeQuent SCB (B. Braun Melsungen) |
| Sirolimus- vs Paclitaxel-Coated Balloon for the Treatment of Coronary In-Stent Restenosis: The SIBLINT-ISR Randomized Trial | 2025 | 258 | SeQuent Please NEO (B. Braun Melsungen) | SeQuent SCB (B. Braun Melsungen) |
| Comparing the Efficacy of Sirolimus and Paclitaxel-Eluting Balloon Catheters in the Treatment of Coronary In-Stent Restenosis: A Prospective Randomized Study (TIS 2 Study) | 2025 | 145 | SeQuent Please (B. Braun Melsungen) | MagicTouch (Concept Medical) |
| Sirolimus-coated versus paclitaxel-coated balloons for bifurcated coronary lesions in the side branch: the SPACIOUS trial | 2025 | 230 | Not publicly available | Not publicly available |
| **Previous observational studies** |  |  |  |  |
| **Title** | **Publication year** | **Nr. patients** | **PCB type (manufacturer)** | **SCB type (manufacturer)** |
| Paclitaxel-coated versus sirolimus-coated balloon angioplasty for revascularization of coronary arteries: the SIRPAC study | 2021 | 1090 | Elutax SV (AR Baltic) | MagicTouch (Concept Medical) |
| Comparison of Sirolimus-Versus Paclitaxel-Coated Balloons in Coronary Artery Disease: One-Year Results of Two Real-World Prospective Registries | 2025 | 2596 | Protégé (Translumina) | MagicTouch (Concept Medical) |
| Sirolimus- Versus Paclitaxel-Coated Balloons for Treatment of Coronary Artery Disease | 2025 | 1320 | Multiple real-world DCB | Multiple real-world DCB |
| **Ongoing randomized controlled trials** |  |  |  |  |
| **Title** | **ClinicalTrials.gov ID** |  |  |  |
| Optimal Treatment for Coronary Drug Eluting Stent In-stent Restenosis (OPEN-ISR) | NCT04862052 |  |  |  |
| ACOART SCB BIF: Treatment of Coronary Bifurcation Lesion by Sirolimus Coated Balloon vs Paclitaxel Coated Balloon | NCT04899583 |  |  |  |
| Efficacy and Safety of Sirolimus-coated Coronary Balloon Dilatation Catheter for De Novo Coronary Bifurcation Lesions | NCT06822712 |  |  |  |
| Efficacy and Safety of Sirolimus-Coated Spiral Balloon for Coronary Bifurcation Lesions | NCT06618248 |  |  |  |
| Sirolimus DEB in Coronary Bifurcation Lesions | NCT04896177 |  |  |  |

PCB = paclitaxel-coated balloon; SCB = sirolimus-coated balloon.

**
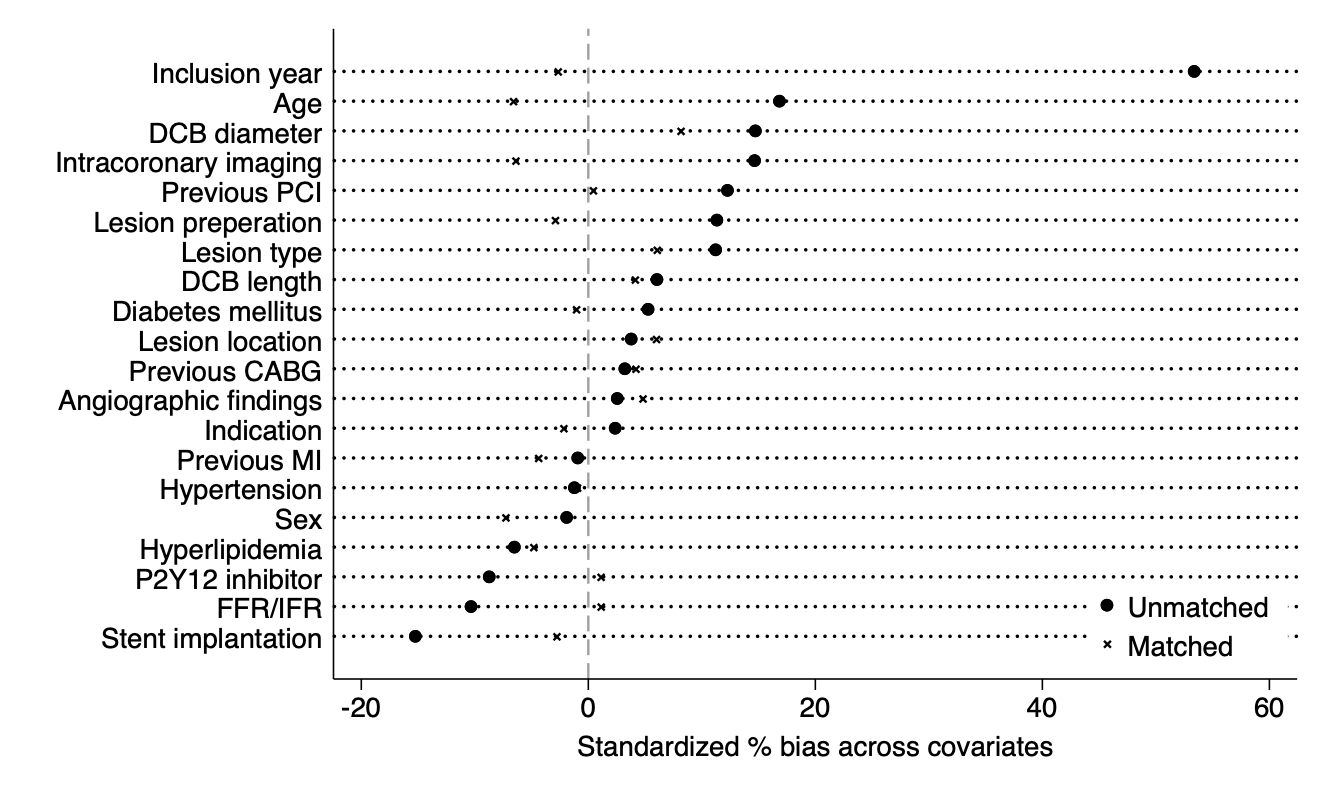
Supplemental Figure S1. Standardized % bias across covariates,** figure showing standardized bias before and after propensity score matching.

CABG = coronary artery by-pass graft; DCB = drug coated balloon; FFR = fractional flow reserve; IFR = instantaneous wave free ratio; MI = myocardial infarction; PCI = percutaneous coronary intervention.


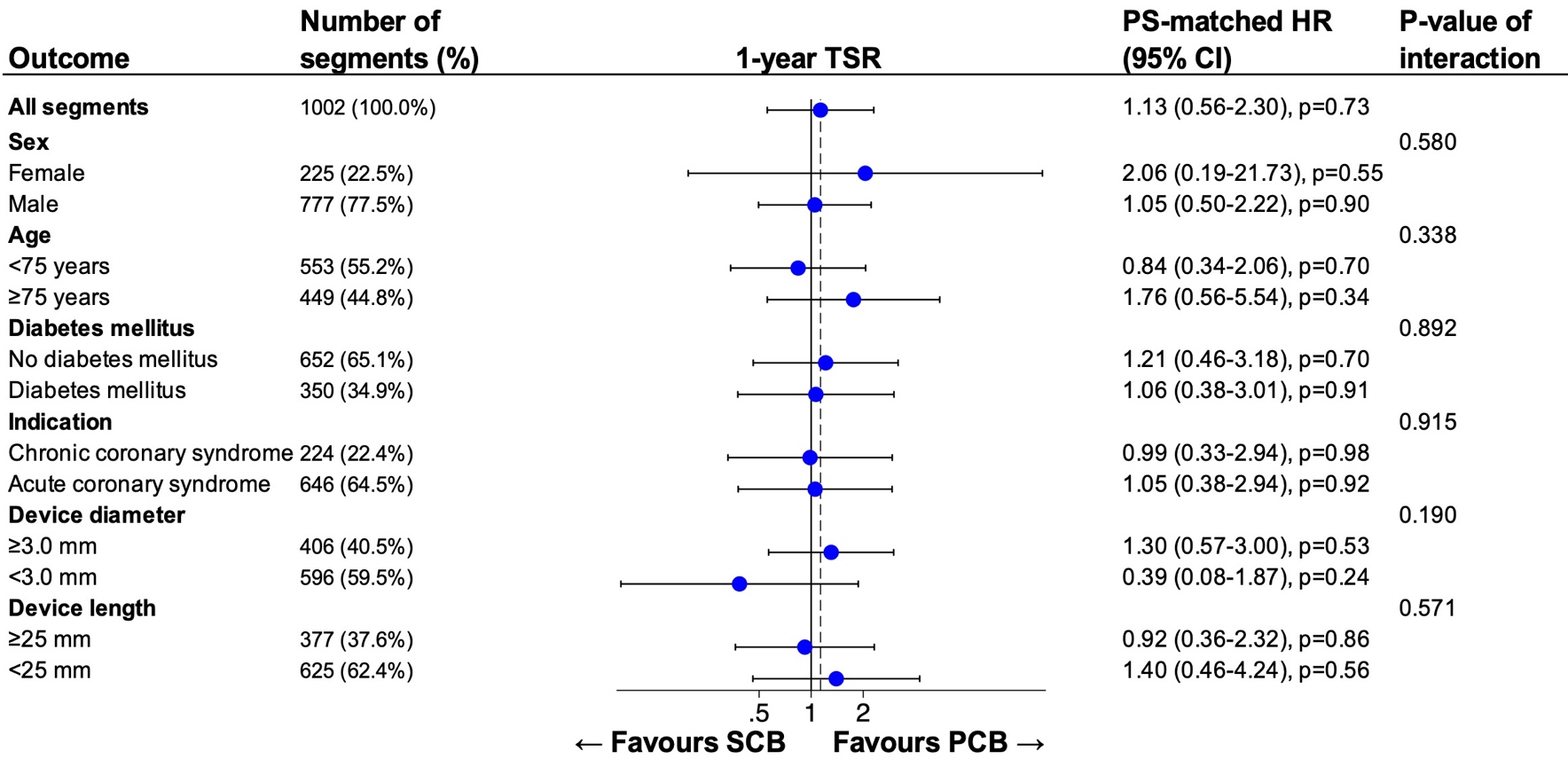


**Supplemental Figure S2. Subgroup Analysis,** forest plot illustrating subgroup analysis for the following subgroups: sex, age, diabetes mellitus, indication, device diameter, and device length. The subgroups were analyzed individually using univariable Cox regression in the PS-matched cohort in terms of 1-year TSR. In addition, interaction test was applied to test for treatment-by-subgroup interactions.

CI = confidence interval; HR = hazard ratio; PCB = paclitaxel-coated balloon; SCB = sirolimus-coated balloon.

**
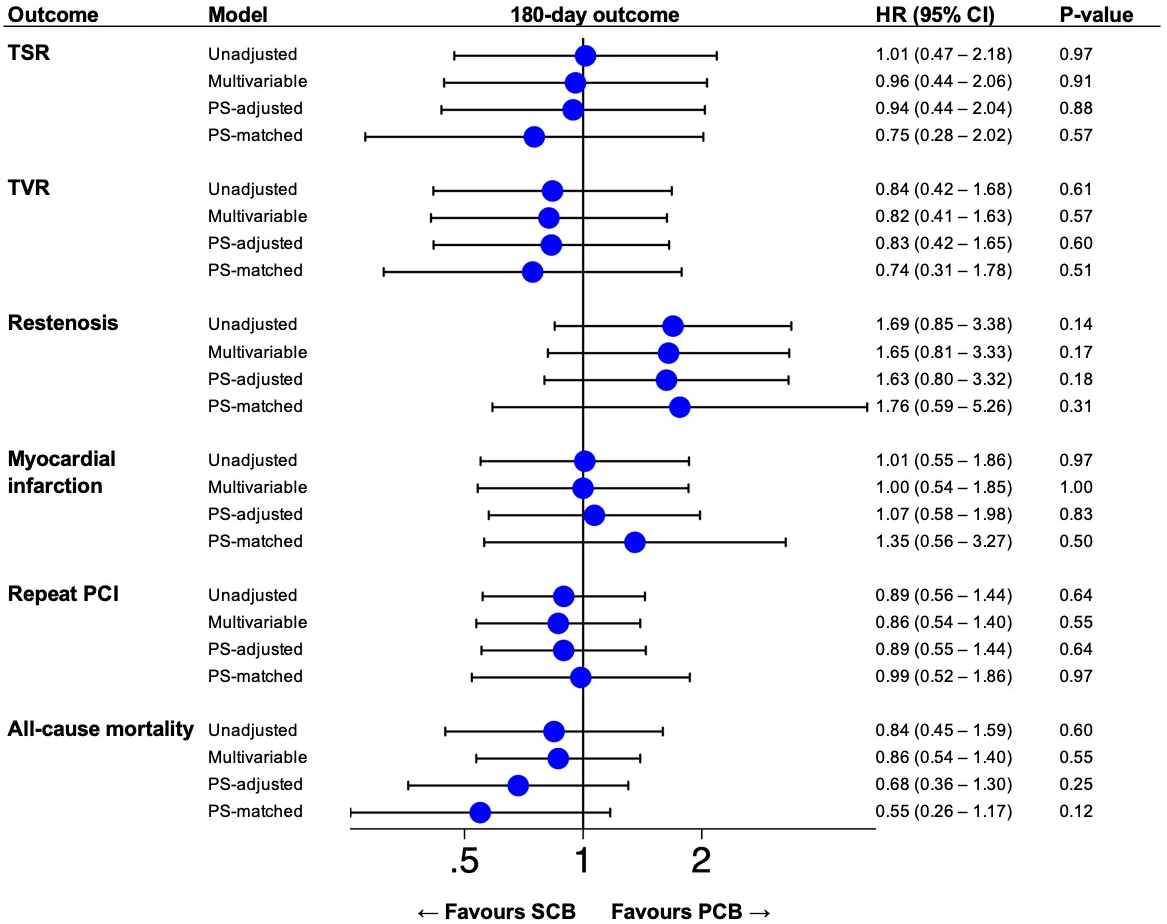
Supplemental Figure S3. 180-Day Outcome,** 180-day outcome of TSR, TVR, restenosis, myocardial infarction, repeat PCI and all-cause mortality. Outcome was assessed using Cox regression across four models: unadjusted, multivariable-adjusted, propensity score-adjusted and propensity score-matched.

CI = confidence interval; HR = hazard ratio; PCB = paclitaxel-coated balloon; PCI = percutaneous coronary intervention; PS = propensity score; SCB = sirolimus-coated balloon; TSR = target segment revascularization; TVR = target vessel revascularization.


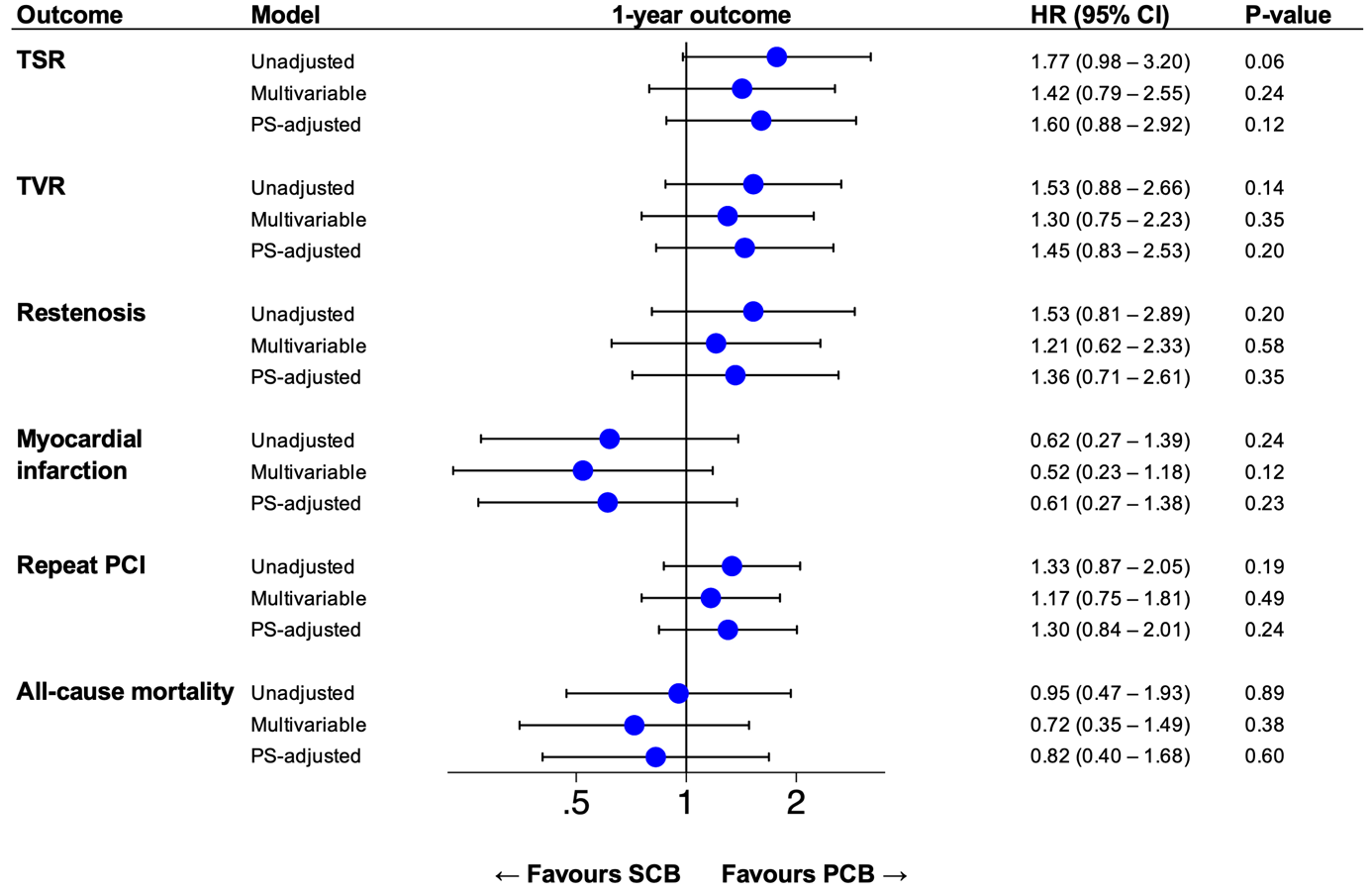


**Supplemental Figure S4, Sensitivity Analysis Restricted to Patients with 1-Year Available Follow-Up,** all outcomes were ascertained up until 16 May 2025**.** Patients included after 16 May 2024 were excluded to ensure 1-year available follow-up for all patients. 1-year outcome was assessed of TSR, TVR, restenosis, myocardial infarction, repeat PCI and all-cause mortality. Outcome was assessed using Cox regression across three models: unadjusted, multivariable-adjusted and propensity score-adjusted.

CI = confidence interval; HR = hazard ratio; PCB = paclitaxel-coated balloon; PCI = percutaneous coronary intervention; PS = propensity score; SCB = sirolimus-coated balloon; TSR = target segment revascularization; TVR = target vessel revascularization.
